# Alexander Waugh

**Published:** 1906-12

**Authors:** 


### ALEXANDER WAUGH, L.R.C.P., M.R.C.S.,
Midsomer Norton.

The death of Dr. Waugh from pneumonia on December 9th, 1906, at the age of 66, is much to be regretted. He was born at Warminster, was educated at Radley College, and then became a student of the Bristol Medical School, where he obtained gold medals for both surgery and medicine; he was also senior prizeman in practical anatomy, and he obtained the Henry Clarke scholarship. He studied also at St. Bartholomew's Hospital, and obtained the M.R.C.S. in 1863 and the L.R.C.P. in 1864. He commenced practice in Midsomer Norton at the age of 24, and has been identified with every side of the life of the town and district for the last 41 years, where he will be long honoured as much for his public services as for his more strictly professional and private ones. For many years the Medical Officer of Health, he was conspicuous in furthering many improvements effected by the Local Board of the neighbourhood. He was the principal founder of the Paulton Cottage Hospital at a time when there was no sort of provision to meet the inevitable accidents in the coal pits of the district, and for 35 years was the moving spirit in the maintenance of that useful institution.

Always a constant attendant at the meetings of the Bath and Bristol Branch of the British Medical Association, he was a deservedly popular president in 1880–81, and at a much earlier age than has been usual. He was for 20 years president of the local cricket club, and had shown a keen love of sport from his early days, when he rowed in the Light at Radley and was in the Rugby XV. In later years he was well known as a splendid shot and fisherman, and he knew more of the habits and *habitats* of wild birds than any man in the county. An ideal country gentleman and a keen politician, he was president of the district Conservative Association, also of the district Choral Society, and of late years no public work in the town was ever attempted without his co-operation and cordial support. He was as popular in private life as in his public one, and his consideration and geniality towards all who came into professional relations with him will long be remembered.

